# Combining biomarkers for prognostic modelling of Parkinson’s disease

**DOI:** 10.1136/jnnp-2021-328365

**Published:** 2022-05-16

**Authors:** Nirosen Vijiaratnam, Michael Lawton, Amanda J Heslegrave, Tong Guo, Manuela Tan, Edwin Jabbari, Raquel Real, John Woodside, Katherine Grosset, Viorica Chelban, Dilan Athauda, Christine Girges, Roger A Barker, John Hardy, Nicholas Wood, Henry Houlden, Nigel Williams, Yoav Ben-Shlomo, Henrik Zetterberg, Donald G Grosset, Thomas Foltynie, Huw R Morris, PRoBaND clinical consortium

**Affiliations:** 1 Department of Clinical and Movement Neurosciences, University College London, UCL Queen Square Institute of Neurology, London, UK; 2 Population Health Sciences, University of Bristol, Bristol, UK; 3 Department of Social Medicine, University of Bristol, Bristol, UK; 4 Dementia Research Institute, University College London, London, UK; 5 Department of Neurodegenerative Disease, UCL Institute of Neurology, Queen Square, London, UK; 6 Department of Neurology, Oslo University Hospital, Oslo, Norway; 7 Aligning Science Across Parkinson’s (ASAP) Collaborative Research Network, Chevy Chase, MD, 20815; 8 Department of Neurology, Southern General Hospital, University of Glasgow and Institute of Neurological Sciences, Glasgow, UK; 9 Cambridge Centre for Brain Repair, University of Cambridge, Cambridge, UK; 10 Molecular Neuroscience, University College London Institute of Neurology, London, UK; 11 MRC Centre for Neuromuscular Diseases, UCL Institute of Neurology and National Hospital for Neurology and Neurosurgery, London, UK; 12 Cardiff University, Cardiff University Institute of Psychological Medicine and Clinical Neurosciences, Cardiff, UK; 13 Clinical Neurochemistry Laboratory, Sahlgrenska University Hospital, Mölndal, Sweden; 14 Department of Psychiatry and Neurochemistry, Institute of Neuroscience and Physiology, the Sahlgrenska Academy at the University of Gothenburg, Mölndal, Sweden; 15 Hong Kong Center, for Neurodegenerative Diseases, Hong Kong, People's Republic of China

**Keywords:** parkinson's disease

## Abstract

**Background:**

Patients with Parkinson’s disease (PD) have variable rates of progression. More accurate prediction of progression could improve selection for clinical trials. Although some variance in clinical progression can be predicted by age at onset and phenotype, we hypothesise that this can be further improved by blood biomarkers.

**Objective:**

To determine if blood biomarkers (serum neurofilament light (NfL) and genetic status (glucocerebrosidase, *GBA* and apolipoprotein E (*APOE*))) are useful in addition to clinical measures for prognostic modelling in PD.

**Methods:**

We evaluated the relationship between serum NfL and baseline and longitudinal clinical measures as well as patients’ genetic (*GBA* and *APOE*) status. We classified patients as having a favourable or an unfavourable outcome based on a previously validated model, and explored how blood biomarkers compared with clinical variables in distinguishing prognostic phenotypes.

**Results:**

291 patients were assessed in this study. Baseline serum NfL was associated with baseline cognitive status. Nfl predicted a shorter time to dementia, postural instability and death (dementia—HR 2.64; postural instability—HR 1.32; mortality—HR 1.89) whereas APOEe4 status was associated with progression to dementia (dementia—HR 3.12, 95% CI 1.63 to 6.00). NfL levels and genetic variables predicted unfavourable progression to a similar extent as clinical predictors. The combination of clinical, NfL and genetic data produced a stronger prediction of unfavourable outcomes compared with age and gender (area under the curve: 0.74-age/gender vs 0.84-ALL p=0.0103).

**Conclusions:**

Clinical trials of disease-modifying therapies might usefully stratify patients using clinical, genetic and NfL status at the time of recruitment.

**Key messages**

- Combining biomarkers could improve clinical trial selection in Parkinson’s disease by better predicting future progression
- Blood biomarkers such as neurofilament light and patient’s genetic status can differentially predict motor and cognitive progression as well as death.
- Combining these blood biomarkers with previously validated clinical markers can provide an excellent prediction of more rapid progression and therefore potentially be used in future trial patient selection

## Introduction

Parkinson’s disease (PD) is a progressive neurodegenerative disorder characterised by a wide range of motor and non-motor features, which results in substantial morbidity.^1^ Disease modification to slow the rate of progression remains a key goal in PD.^2^ A challenging aspect is the inherently complex nature of PD with substantial clinical heterogeneity in the rate of progression.^1 3^ The underlying basis for this variability is poorly understood but may relate to cellular susceptibility, inflammation, cell to cell spread of pathogenic proteins and compensatory mechanisms.^4^ Ultimately, this likely relates at least in part to genetic variation^5^ though findings have been inconsistent.^6 7^ The strongest candidates noted are the E4 allele of apolipoprotein E (APOE) and glucocerebrosidase (GBA) mutations.

APOE-E4 affects progression to cognitive decline in PD^7 8^ as do GBA mutations although the risk of development of dementia in GBA mutation carriers varies based on the type of mutation^9^ while their impact on motor progression is less clear.^7 10^ The impact of these genetic factors on overall survival has also been studied though findings are inconsistent.^11 12^

Neurofilament light (NfL) is a neurofilament subunit. Neurofilaments are structural proteins that confer stability to neurons and are expressed abundantly in larger myelinated axons.^13^ NfL is constantly released into cerebrospinal fluid (CSF) and subsequently blood, with levels increasing in response to axonal injury thus making peripheral measurement of NfL a potentially useful biomarker of a range of CNS diseases.^13^ Despite its lack of specificity, the association of NfL with axonal injury and the amount of neuronal damage means that it may be useful in predicting progression and survival in several neurodegenerative diseases including PD.^14–17^

Unbalanced randomisation in clinical trials can have a significant effect on the power of the study to detect the impact of an intervention.^18^ Investigating the reliability of NfL alone and in combination with patients’ genetic status may form a critical aspect in prognostic prediction which will be important for patient selection in future PD clinical trials. We formally explored this hypothesis in a large prospectively followed cohort of patients with a recent diagnosis of PD. We determined if baseline NfL levels related to the severity of symptoms soon after diagnosis and with genetic status. We then explored whether NfL and genetic status predicted subsequent motor and cognitive progression and survival. The potential use of NfL alone and in conjunction with clinical outcomes and genetic status in improving clinical progression modelling for use in clinical trial selection was then explored with the overall hypothesis being that the combination of blood biomarkers with previously validated clinical variables would improve the distinction between patients with a favourable or unfavourable prognosis.

## Methods

### Participants

PD participants in this study were recruited from the Tracking Parkinson’s study, a large prospective, observational, multicentre project which recruited patients from 1 February 2012 to 31 May 2014. The study protocol and baseline patient characteristics have been published.^19^ Briefly, patients with a clinical diagnosis of PD meeting the Queen Square Brain Bank criteria^20^ and supportive neuroimaging (when the diagnosis was not firmly established clinically) were enrolled. Patients had to be within 3.5 years of diagnosis at recruitment. Both drug-naïve and treated patients aged 18–90 years were eligible. Exclusion criteria were severe comorbid illness that precluded clinic visits, and other degenerative forms of parkinsonism. Patients were excluded from further follow-up if their diagnosis was revised to an alternative condition.

Patients were selected for NfL analysis based on completion of a minimum follow-up of 2.5 years, with available serum samples at baseline for analysis. Further selection criteria were also applied to facilitate an analysis of whether NfL might help discriminate typical PD with a high index of diagnostic certainty (>95%), from an equivalent sample of cases with atypical clinical features with a lower index of diagnostic certainty (<80%) at their 2.5-year clinical assessment.

### Clinical assessments

Baseline demographics such as gender, age and disease duration were recorded. A detailed description of clinical assessments performed in Tracking Parkinson’s has previously been published.^19^ In this study, we included selective motor (Movement Disorders Society Unified Parkinson’s Disease Rating Scale part 3—MDS-UPDRS3 & Hoehn & Yahr—H&Y), cognitive (Montreal Cognitive Assessment—MoCA, Animal Semantic Fluency Score—SF), functional (Schwab and England) and quality of life (PD Questionnaire-8) measures. All patients had been diagnosed within the preceding 3.5 years of study entry and a proportion underwent assessments every 18 months (although there were some interim visits at 6–12 months intervals which collected other information) with data available up to visit 10 (72 months) for this study. Clinicians determined their diagnostic certainty of PD at each visit (0%–100%), while also noting clinical features they deemed to be atypical for PD. Patients who received an alternative diagnosis to PD during follow-up or who had a clinician diagnostic certainty of <90% at the last available visit were excluded from this analysis. All-cause mortality was also noted and studied as a relevant outcome.

### Favourable versus unfavourable outcome subgroups

Patients were classified as having favourable or unfavourable outcomes based on a previously validated model of progression.^21^ A binary outcome measure was created for unfavourable progression PD (U-PD) when patients had postural instability (defined by a H&Y scale score of 3 or higher) or dementia (defined by adapted Movement disorders society criteria for PD dementia (MOCA <21 and impairment in at least 2 domains, cognitive deficits impacting on daily living—MDS UPDRS 1.1≥2 and no severe depression—MDS UPDRS 1.3<4)^8^ at the last available assessment, or if they had died during follow-up. Although the premise for grouping was identical to the previously validated model, our definition of dementia varied (level 1 criteria from the Movement Disorder Society Task Force and operationalised using The Mini-Mental State Exam (MMSE) and either clock drawing or phonemic fluency tests was used in the model development study^22^). All other patients were classified as having favourable progression PD (F-PD). Patients already demonstrating U-PD characteristics at baseline were excluded from the progression to U-PD analyses, but were retained in the baseline analysis and the mixed effects regression analysis.^21^ The three baseline variables (age at baseline, MDS-UPDRS axial score and animal SF) that were previously identified to predict the development of U-PD^21^ were then explored individually and in combination with NfL and patients genetic status to compare clinical, genetic and biomarker data in predicting progression.

### Sample collection and measurement

At enrolment, 10 mL of venous blood was collected from each participant in serum separator tubes. Blood samples were centrifuged (2500 g for 15 min) within 1 hour of collection. Serum aliquots were stored in cryotubes at −80°C. Serum NfL concentration was measured using the NF-Light Advantage kit on the HD-X Analyzer (Quanterix, Billerica, Massachusetts, USA) by researchers who were blinded to the clinical diagnosis, as previously described.^23^ Full details are available on protocols.io: https://dx.doi.org/10.17504/protocols.io.bzbep2je.^24^

### Genetic status classification

Molecular genetic analysis techniques for determining patients *APOE* and *GBA* status have previously been described.^7 25^ The step-by-step protocol for SNP genotyping and *APOE* genotyping is available on protocols.io: https://dx.doi.org/10.17504/protocols.io.by9ypz7w.^26^

As we and others have previously identified, *APOE* ε4 status is known to be a determinant of cognitive progression, thus patients were classified into groups of either being ε4 carriers (homozygous and heterozygous) and non-carriers.^7^ Mutations identified and classification approaches for determining *GBA* prognostic status in the Tracking Parkinson’s study have previously been detailed.^25^ A step-by-step protocol for *GBA* genotyping is available on protocols.io: https: dx.doi.org/10.17504/protocols.io.bzd7p29n.^27^ Patients in this study were classified into groups where a *GBA* variant was identified as either being pathogenic in Gaucher disease (GD) and associated with PD in the heterozygous state (GD causing) (L444P (5 cases), p.R463C (1 case), p.R395C (1 case), p.G377S (1 case), p.N370S (1 case) and p.D409H/L444P/A456P/V460V (1 case)) or non-synonymous genetic variants that are associated with PD (non-GD causing) (E326K (10 cases), T369M (7 cases) and p.D140H/p.E326K (1 case). Two cases with variants of unknown significance were excluded from the group analysis (p.M123T, p.R262H).

### Statistical analysis

Descriptive statistics including mean, SD, median, IQR, frequencies and percentages were used to describe demographic and clinical characteristics by groups. Given non-normally distributed data, differences were compared using Kruskal-Wallis tests for continuous data and χ^2^ tests for categorical data. A Natural logarithm (Ln) transformation was performed to reduce right skewness for NfL levels as indicated by inspection of residuals.

Univariate and multivariable (adjusting for age, gender and disease duration) linear regression analysis was performed to investigate the association between baseline NfL levels and clinical measures of PD at baseline. The interaction between *GBA* and *APOE* status with NfL was explored with univariate and multivariate linear regression with NfL as the outcome measure and the respective positive gene status being compared with those who were negative.

Associations between baseline serum NfL levels and genetic status and change in motor, cognitive and quality of life outcomes over time (disease duration from diagnosis as the time axis) were then investigated by linear mixed effects analysis, adjusted for age at diagnosis and gender. The mixed models had both a random intercept and a random slope. Cox proportional hazards regression was then used to investigate whether the baseline NfL level and genetic status individually and when combined predicted, postural instability, dementia and mortality after adjustment for age, gender and baseline MDS-UPDRS 3.

Logistic regression was repeated using previously validated baseline predictive clinical variables (MDS-UPDRS axial score and SF) individually and in combination with NfL levels, and the patients’ *GBA* and *APOE* status to explore the ability to distinguish predetermined outcome groups (U-PD vs F-PD). The area under the curve (AUC) for each combination of variables was statistically compared against NfL alone, and together with NfL using Delong’s test.

The Youden J index (maximum sensitivity +specificity – 1) was then calculated for all points of the receiver operator characteristic curve and the maximum value of the index was used as a criterion for selecting the optimum NfL cut-off point for distinguishing U-PD and F-PD. All tests were two-sided. All statistical analysis and figures were generated using RRID:SCR_012763, version 16.1.

### Data and code availability

The original data used in this study is available from the Tracking Parkinson’s (www.trackingparkinsons.org.uk) team. The analysis protocol and code are available at GitHub (https://github.com/huw-morris-lab/proband-nfl) and Zenodo (doi: https://doi.org/10.5281/zenodo.5525370)

## Results

**Figure 1:**
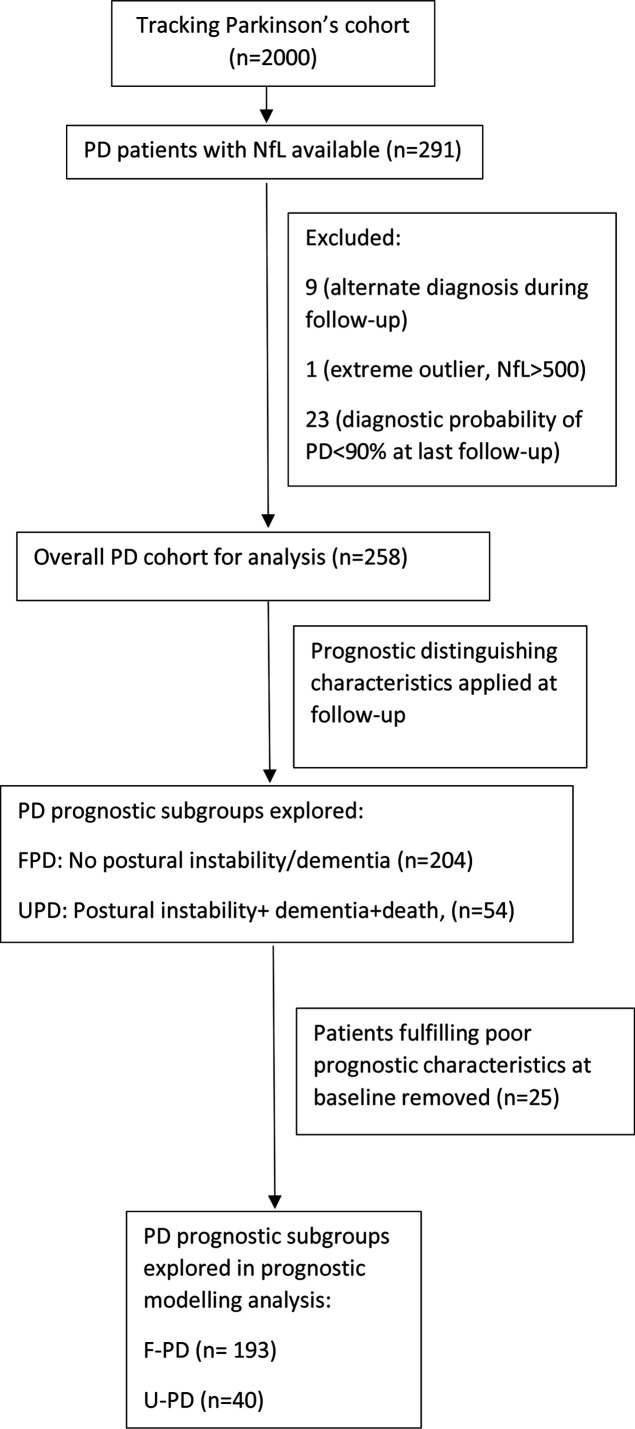
Summary of study design. F-PD, favourable progression PD; Nfl, neurofilament light; PD, Parkinson’s disease; U-PD, unfavourable progression PD.

Of the 2000 patients enrolled into the Tracking Parkinson’s study, 291 were studied based on selection criteria. The demographic (age, gender, disease duration from diagnosis) and baseline clinical characteristics (MDS UPDRS 3, H&Y and MOCA) of this cohort was similar to the remaining cohort (online supplemental table 1). The purpose of this selection approach was to provide good representation of a subset of cases to model progression and to explore the possible use of baseline NfL to determine conversion to an atypical parkinsonian syndrome, in an early Parkinsonism cohort. The number of rediagnosed cases was however low: including three cases of progressive supranuclear palsy, one multiple system atrophy and five with other diagnosis (one postpolio syndrome, one vascular parkinsonism, one parkinsonism with a scan without evidence of dopaminergic deficit, one essential tremor and one uncertain diagnosis) and these cases were excluded from further analysis in addition to a case which was deemed an outlier (NfL >2.5 times above cohort mean) and cases with a PD diagnostic certainty of <90% at the last available visit (figure 1). Progression and phenotype analysis was then performed on the remaining 258 patients. Of these cases, 252 were assessed at 18 months while 217 128 and 60 were assessed at 36, 54 and 72 months, respectively.

10.1136/jnnp-2021-328365.supp1 Supplementary data

### Evaluation of the relationship between NFL, clinical features and genetic status at baseline

PD participant demographics and clinical features at baseline are summarised in table 1. Serum NfL concentrations were associated with age (Coefficient=5.86, p<0.001) but not gender or disease duration. Baseline MoCA and SF scores were significantly associated with serum NfL levels (MoCA Coefficient −0.60, p=0.021; SF Coefficient −1.77, p=<0.001), indicating that serum NfL is associated with baseline markers of cognitive impairment. This remained significant for SF after adjustment for age, gender and disease duration. NfL was not associated with measures of functional status at baseline, nor with motor symptom severity measured by the H&Y, MDS-UPDRS 3 total and subscores (rigidity, bradykinesia, axial and tremor) (table 1). There was no significant association between NfL levels and *GBA* or *APOE* status (table 1).

**Table 1:** Evaluation of the relationship between NFL and clinical features of PD at baseline

Variables	Mean (SD) or total (%)	Univariate, coefficient (95% CI)	P value	Multivariate, coefficient (95% CI)	P value
Age at baseline	68.4 (8.9)	5.86 (4.85 to 6.86)	<0.001		
Disease duration from diagnosis	1.3 (0.9)	0.07 (−0.05 to 0.20)	0.240		
Gender, male (%)	165 (63.7)	0.05 (−0.20 to 0.13)	0.692		
Genetic status					
*GBA*-positive (non-GD variant)	18/240 (7.5)	0.14 (−0.37 to 0.65)	0.590	0.30 (−0.12 to 0.72)	0.155
*GBA*-positive (GD variant)	10/240 (4.2)	−0.45 (−0.37 to 0.65)	0.590	0.02 (−0.51 to 0.55)	0.945
*APOE* ε4 heterozygous	63/236 (26.7)	−0.19 (−0.48 to 0.09)	0.186	0.09 (−0.14 to 0.33)	0.433
*APOE* ε4 homozygous	8/236 (3.4)	0.34 (−0.37 to 1.04)	0.350	0.52 (−0.04 to 1.08)	0.07
Motor severity outcomes					
H&Y	1.8 (0.6)	0.08 (−0.01 to 0.16)	0.068	0.01 (−0.09 to 0.11)	0.835
MDS-UPDRS 3 total	22.8 (11.6)	−0.73 (−2.37 to 0.91)	0.382	−1.80 (−3.82 to 0.22)	0.080
MDS-UPDRS rigidity	3.8 (2.9)	−0.35 (−0.76 to 0.05)	0.085	−0.43 (−0.92 to 0.07)	0.092
MDS-UPDRS bradykinesia	10.9 (7.0)	−0.46 (−1.44 to 0.52)	0.354	−0.80 (−2.01 to 0.42)	0.197
MDS-UPDRS axial	2.9 (2.6)	0.34 (−0.03 to 0.70)	0.069	−0.01 (−0.03 to 0.44)	0.961
MDS-UPDRS tremor	4.3 (4.0)	−0.37 (−0.94 to 0.19)	0.190	−0.66 (−1.36 to 0.03)	0.062
Cognitive outcomes					
MoCA	25.1	−0.60 (−0.04 to 0.00)	**0.021**	−0.38 (−1.01 to 0.25)	0.236
Semantic fluency	21.2	−1.77 (−2.63 to 0.92)	**<0.001**	−**1.10** (**−2.16 to 0.04**)	**0.043**
Functional outcomes					
SEADL	86.3 (11.7)	−0.53 (−2.18 to 1.11)	0.524	0.57 (−1.48 to 2.61)	0.587
PDQ8	6.3 (4.8)	−0.32 (−1.01 to 0.36)	0.353	0.41 (−0.41 to 1.23)	0.327

Univariate and multivariable (age at baseline, gender and disease duration) linear regression analysis on baseline NfL with baseline clinical measures in PD patients treated as outcome measures. In regression analysis of NfL and genetic status, NfL was treated as the outcome measure and patients who were positive for a genetic mutation were compared with those who were not.

Values in bold demarcate statistical significance

APOE, apolipoprotein E; GBA, Glucocerebrocidase; H&Y stage, Hoehn and Yahr stage; MDS-UPDRS, Movement Disorders Society Unified Parkinson’s disease rating scale; MoCA, Montreal Cognitive Assessment; NfL, Neurofilament light protein; PD, Parkinson’s disease; PDQ8, Park’nson’s Disease Questionnaire-8; SEADL, Schwab and England scale.

### Evaluation of biomarker prediction of PD progression and mortality

We explored the ability of baseline genetic status and NfL to predict motor, cognitive and functional progression with mixed effects linear models. The MDS-UPDRS 3 score increases with increasing motor impairment, whereas the SEADL decreases with increasing functional impairment. In our analysis of the rate of change of the MDS-UPDRS, a significant negative association with the intercept was noted between baseline NfL and patients overall (total MDS-UPDRS 3 coefficient −3.55, p=0.001) and subsection (rigidity, bradykinesia, axial and tremor) motor scores. A similar association was also noted with patients' overall functional status (SEADL Coefficient 3.36, p=0.004). There was no association between the intercept for cognitive or quality of life scores and NfL. Baseline serum NfL was associated with a more rapid overall progression of motor PD features (as assessed using the total MDS-UPDRS 3, coefficient 0.79, p=0.012) as well as those thought to be more reflective of underlying disease progression using subsection motor scores of the UPDRS (UPDRS axial, bradykinesia, rigidity) and the H&Y scores, 0.06, p=0.001 (table 2). Baseline serum NfL was not significantly associated with the changes in cognition scores (MoCA and SF), though higher levels of NfL at baseline predicted a faster rate of worsening overall function (SEADL Coefficient-1.51, p<0.001). Baseline *GBA* status did not predict progression of any of the measures while *APOE* status predicted a more rapid cognitive decline (MOCA Coefficient −0.43, p<0.001) (table 2).

**Table 2:** Relationship between baseline NFL level, GBA and APOE status and change in motor, cognitive and functional scores using linear mixed effects models

Variable	Main effect, coefficient – Intercept (95% CI), p value			Interaction with time–slope coefficient (95% CI), p value		
	NfL	*GBA*	*APOE*	NfL	*GBA*	*APOE*
H&Y	−0.11 (–0.23 to 0.01), 0.061	0.02 (–0.18 to 0.21, 0.880	−0.12 (–0.28 to 0.04), 0.151	0.06 (0.02 to 0.08), **0.001**	0.00 (–0.06 to 0.06), 0.967	0.02 (–0.02 to 0.07), 0.335
MDS-UPDRS 3 Total	−3.55 (–5.68 to –1.43), **0.001**	−0.25 (–1.03 to 0.92), 0.218	−2.48 (–5.46 to 0.51), 0.104	0.79 (0.17 to 1.43), **0.012**	−0.05 (–1.03 to 0.92), 0.912	0.69 (–0.17 to 1.56), 0.116
MDS-UPDRS Rigidity	−0.82 (–1.38 to –0.25), **0.004**	−0.71 (–1.65 to 0.23), 0.140	−0.74 (–1.52 to 0.04), 0.062	0.20 (0.04 to 0.36), **0.016**	0.10 (–0.18 to 0.37), 0.491	0.19 (–0.04 to 0.42), 0.104
MDS-UPDRS Bradykinesia	−1.87 (–3.14 to –0.60), **0.004**	−1.46 (–3.57 to 0.65), 0.175	0.29 (–1.52 to 2.10), 0.752	0.42 (0.07 to 0.77), **0.019**	0.03 (0.54 to 0.60), 0.912	0.00 (–0.47 to 0.48), 0.989
MDS-UPDRS Axial	−1.20 (–1.94 to –0.46), **0.002**	0.57 (–0.62 to 1.75), 0.348	−0.96 (–1.98 to 0.06), 0.065	0.38 (0.06 to 0.70), **0.018**	−0.38 (–0.06 to 0.12), 0.138	0.41 (–0.03 to 0.85), 0.07
MDS-UPDRS Tremor	−0.79 (–1.54 to –0.01), **0.046**	−0.09 (–1.20 to 1.38), 0.894	−1.11 (–2.21 to 1.38), 0.048	0.05 (–0.13 to 0.22), 0.588	−0.28 (–0.56 to 0.01), 0.060	0.15 (–0.10 to 0.40), 0.241
MoCA	0.07 (–0.56 to 0.69), 0.839	−0.14 (–1.11 to 0.83), 0.775	−0.47 (–1.27 to 0.34), 0.258	−0.17 (–0.34 to 0.01), 0.062	0.14 (–1.32 to 0.41), 0.312	−0.43 (–0.66 to –0.19), <**0.001**
Semantic Fluency	−0.61 (–1.68 to 0.46), 0.263	2.26 (0.54 to 3.98), 0.010	−0.90 (–2.41 to 0.61), 0.243	−0.03 (–0.31 to 0.24), 0.803	−0.37 (–0.81 to 0.07), 0.100	−0.34 (–0.71 to 0.04), 0.077
SEADL	3.36 (1.08 to 5.64), **0.004**	−0.02 (–3.82 to 3.78), 0.991	1.55 (–1.59 to 4.68), 0.333	−1.51 (–2.30 to –0.72), <**0.001**	0.09 (−1.26 to –1.44), 0.899	−0.49 (–1.58 to 0.59), 0.373
PDQ8	0.02 (–0.86 to 0.89), 0.970	−0.46 (–1.87 to 0.96), 0.527	−0.14 (–1.38 to 1.10), 0.824	0.06 (–0.16 to 0.284), 0.616	−0.02 (–0.37 to 0.33), 0.910	−0.05 (–0.36 to 0.27), 0.762

Linear mixed effects analysis on baseline NfL levels, GBA and APOE status with clinical outcomes in PD patients over time adjusted for age at diagnosis and gender. The main effect indicates the effect of the assessed baseline variable on the intercept and the interaction with time indicates the effect on the slope (change in value per year) of the model. The GBA group includes GD causing and non-GD causing mutation carriers; the APOE group includes APOEe4 heterozygous and homozygous carriers.

Values in bold demarcate statistical significance

APOE, apolipoprotein E; GBA, Glucocerebrosidase; GD, Gaucher disease; H&Y, Hoehn and Yahr stage; MDS-UPDRS, Movement Disorders Society Unified Parkinson’s Disease Rating Scale; MoCA, Montreal Cognitive Assessment; NfL, Neurofilament light protein; PD, Parkinson’s disease; PDQ8, Parkinson’s Disease Questionnaire-8; SEADL, Schwab and England scale.

**Figure 2:**
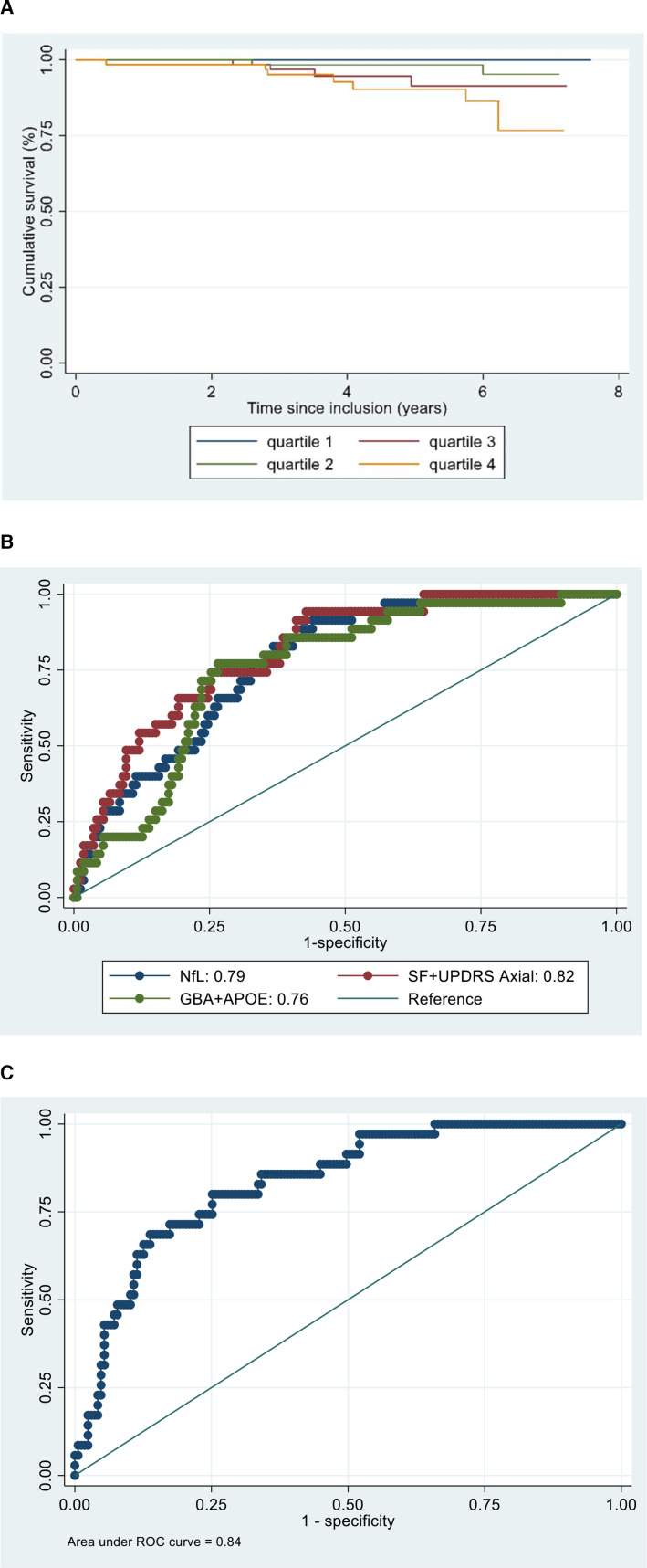
(A) Kaplan-Meir survival estimates by NfL quartiles and receiver operator characteristic curves of (B) individual biomarker components and (C) all biomarker components combined for predicting unfavorable progression. APOE, apolipoprotein E; GBA, glucocerebrosidase; NfL, neurofilament light; SF, semantic fluency; UPDRS, Unified Parkinson’s Disease Rating Scale.

We then explored if baseline genetic status and NfL could predict progression to postural instability, dementia and death using cox regression analysis (table 3). Of the 258 patients studied, 93 developed postural instability over a mean follow-up interval of 3.27 years (SD 1.61). Thirty-five of the 258 patients (13.6%) developed dementia over an average interval of 3.70 years (SD 1.78) while 13 patients (5.0%) died during follow-up (mean 4.87±SD 1.52 years). A higher NfL concentration at baseline predicted a shorter progression to dementia, HR 2.50 (95% CI 1.72 to 3.65), p<0.001). This remained significant following multivariate analysis 2.64 (95% CI 1.58 to 4.41, p<0.001). Similarly, higher baseline NfL concentrations predicted a more rapid progression to postural instability, (Univariate HR 1.50, 95% CI 1.24 to 1.81, p<0.001), Multivariate HR 1.32, 95% CI 1.03 to 1.69, p=0.030). A higher NfL concentration at baseline predicted a shorter survival, HR 1.94 (95% CI 1.36 to 2.76, p<0.001). This remained statistically significant when corrected for age, and gender and baseline MDS-UPDRS 3 (HR 1.89, 95% CI 1.14 to 3.11, p=0.013). The highest baseline NfL quartile conferred a twofold higher risk of mortality in comparison to the lowest quartile (HR 2.04, 95% CI 1.13 to 3.69, p=0.018) (figure 2A).

**Table 3:** Relationship between baseline NFL levels, *GBA* and *APOE* status alone and in combination and the development of dementia, postural instability and death using Cox regression

Variables	Baseline status	HR (95% CI)			
		Univariate	P value	Multivariate	P value
Postural instability	NfL	1.50 (1.24 to 1.81)	**<0.001**	1.32 (1.03 to 1.69)	**0.030**
	*GBA*	0.76 (−0.42 to 1.39)	0.378	1.03 (0.54 to 1.98)	0.927
	*APOE*	0.83 (0.52 to 1.33)	0.443	0.98 (0.61 to 1.57)	0.920
Dementia	NfL	2.50 (1.72 to 3.65)	**<0.001**	2.64 (1.58 to 4.41)	**<0.001**
	*GBA*	0.54 (0.16 to 1.88)	0.337	0.60 (0.15 to 2.38)	0.471
	*APOE*	2.08 (1.16 to 3.73)	**0.014**	3.12 (1.63 to 6.00)	**0.001**
Death	NfL	1.94 (1.36 to 2.76)	**<0.001**	1.89 (1.14 to 3.11)	**0.013**
	*GBA*	1.66 (0.71 to 3.86)	0.241	2.66 (1.04 to 6.79)	**0.041**
	*APOE*	0.43 (0.10 to 1.79)	0.246	0.79 (0.19 to 3.25)	0.744

Univariate and multivariable (age at baseline, gender and MDS-UPDRS three score at baseline) Cox regression analysis on baseline NfL, *GBA* and *APOE* status with progression to dementia and postural instability at the last available visit and death treated as outcome measures. The GBA group includes GD causing and non-GD causing mutation carriers; the APOE group includes APOEe4 heterozygous and homozygous carriers.

Values in bold demarcate statistical significance

APOE, apolipoprotein E; GBA, glucocerebrosidase; GD, Gaucher disease; MDS-UPDRS, Movement Disorders Society Unified Parkinson’s Disease Rating Scale; NfL, neurofilament light protein.

Patients’ *GBA* status did not predict progression to dementia though their *APOE* ε4 status did (Univariate HR2.08, 95% CI 1.16 to 3.73, p=0.014, Multivariate HR 3.12, 95% CI 1.63 to 6.00, p=0.001). GBA and *APOE* ε4 status did not predict progression to postural instability. Although GBA status predicted survival when corrected for baseline age, gender and MDS-UPDRS 3 (HR 2.66, 95% CI 1.04 to 6.79, p=0.041), *APOE* ε4 status did not (table 3).

In modelling combining all biomarkers with baseline age, gender and MDS-UPDRS 3, only *APOE* ε4 status (HR 2.75, 95% CI 1.44 to 5.24, p=0.002) and Nfl (HR 2.09, 95% CI 1.16 to 3.76, p=0.014) continued to significantly predict progression to dementia. Only NfL levels predicted progression to postural instability (HR 1.44, 95% CI 1.04 to 2.01, p=0.029) in the model with all variables combined. NfL levels predicted survival (HR 2.18, 95% CI 1.17 to 4.05, p=0.014) while a trend towards *GBA* status predicting survival (HR 2.33, 95% CI 0.92 to 5.95, p=0.076) was noted.

### Evaluation of biomarker use in progression modelling

We applied distinction criteria (summarised in figure 1) for determining a poor prognosis at the last available follow-up to separate patients into two groups (U-PD and F-PD). PD patients with an U-PD had higher serum NfL levels at baseline than those with a F-PD (41.9 (SD 21.7) vs 29.6 (SD 36.6), p<0.001). Baseline NfL levels were able to distinguish these phenotypes with an AUC of 0.79, 95% CI 0.72 to 0.85 (figure 2B). An optimal cut-off value of 29.0 ng/L was determined by the J Youden index with a sensitivity of 65.0% and specificity of 65.6%.

Baseline variables (MDS-UPDRS axial score, SF and NfL) explored in logistic regression individually and in combination with age at the baseline assessment and gender as covariates are summarised in online supplemental table 3). The AUC for models incorporating variables individually were SF (0.78, 95% CI 0.71 to 0.85), MDS-UPDRS axial (0.79, 95% CI 0.71 to 0.86) and combined genetic status (0.76, 95% CI 0.68 to 0.84). An AUC of 0.82 (95% CI 0.74 to 0.88) was noted in the model combining SF and MDS-UPDRS axial scores. The AUC for this model did not significantly differ from the model with NfL alone (0.79 vs 0.82, p=0.3073) or combined genetic markers (0.76 vs 0.82, p=0.1098) (figure 2B). The addition of NfL to clinical markers did not result in a significant improvement in comparison to clinical markers alone (AUC 0.82 vs 0.85, p=0.1691). The combination of NfL with both clinical markers did however result in a higher AUC for distinguishing PD progression phenotypes in comparison to NFL alone (0.79 vs 0.85, p=0.0163) (online supplemental table 4). The addition of patient’s combined genetic status and baseline NfL levels to clinical variables in the model resulted in an AUC of 0.84 (figure 2C). This combination resulted in a significantly higher AUC for distinguishing progression phenotypes in comparison to age and gender (0.74 vs 0.84, p=0.0121) (table 4). The model combining all markers (MDS-UPDRS axial, SF, NfL, *APOE* and *GBA* status resulted in a similar AUC to models incorporation both clinical variables and NfL with either genetic status (AUC 0.84 vs 0.85) (online supplemental table 4).

**Table 4:** Summary of ROC analysis for models combining baseline predictive variables and comparison of models against model with age and gender

	AUC (95% CI)	P value
Age +gender	0.74 (0.67 to 0.82)	
Genetic status	0.76 (0.68 to 0.84)	0.4712
Genetic status +NfL	0.80 (0.74 to 0.87)	0.0364
Genetic status +NfL+ clinical variables	0.84 (0.78 to 0.91)	0.0103

All models incorporate age and gender as covariates. AUC of each model is compared with age +gender.

AUC, area under the curve; NfL, Neurofilament light protein; ROC, receiver operator characteristic curve.

## Discussion

In this study, we explored the use of serum NfL and candidate genetic variables as potential prognostic biomarkers in a large and well-studied cohort of recently diagnosed PD patients with prolonged follow-up and high clinical diagnostic certainty. We found baseline NfL to be associated with age and aspects of cognition. We also established that serum NfL in combination with genetic variables (*APoE* and *GBA* status) and previously validated clinical measures can provide a better prediction of several aspects of PD progression in prognostic modelling, then clinical measures alone.

Serum NfL is higher in older PD patients. This presumably relates to increased axonal degeneration and decreased clearance that occurs with ageing.^28 29^ If NfL is used as a diagnostic and/or prognostic tool then age adjusted/corrected measures are required.

Considering NfL alone, we did not find an association between NfL and baseline motor severity measures (MDS-UPDRS-3 and H&Y) though a trend towards significance was noted. The significance of association between the MDS-UPDRS 3 (total and subscores) and NfL has varied between studies. A potential explanation for this could be the discrepant use of ‘ON’, ‘OFF’ and treatment naive UPDRS scores. In our study 234 of the 258 cases studied were assessed in the ON state only thus making correction for this of limited value. The association of H&Y status and NfL appears to be more consistent in studies.^16 17 30^ This is potentially attributed to the H&Y stages more prominently reflecting the patient’s axial status at higher levels (>2.5) which seems to better correlate with NfL while also being related to reduction in white matter integrity in the substantia nigra.^31^ The lack of significant association between H&Y and NfL at baseline in our cohort is likely a reflection of the minimal representation of patients with more severe H&Y scores at this assessment time point.

We found that baseline MoCA and SF scores were inversely associated with NfL levels. This finding is consistent with other studies exploring global cognitive function. The association between SF and NfL noted is consistent with a previous study that explored this particular cognitive subdomain.^32^ A deficit in this test is a reflection of fronto-temporal dysfunction.^33^ Abnormalities in axonal tracts in these regions have been noted in the early stages of PD and seem to correlate with CSF NfL levels.^17^ This finding potentially highlights the value of more detailed neuropsychological testing, but this is of course more labour intensive than a simple blood test.

Despite a previous study suggesting higher blood NfL levels in patients with more pathogenic variants of *GBA*,^34^ we did not replicate this finding. Furthermore, we did not note significant differences in NfL levels when comparing patients with a heterozygous or homozygous *APOE* ε4 status to those who did not. These genetic markers are of interest considering their variable association with more severe cognitive and motor progression.^7 10^ We have however confirmed the predictive capacity of APOE ε4 status on cognitive progression and development of dementia,^7 8^ while the lack of impact of GBA variants on motor and cognitive progression in our study compared with previous publications^9 10^ is likely explained by the relatively short duration of follow-up and by the small number of patients in this cohort.

We found that serum NfL levels could predict progression of motor, and functional status while also predicting mortality in PD. We noted a negative main effect of higher baseline NfL levels on progression scores in mixed modelling. This is potentially consistent with NfL levels peaking prior to the onset of appreciable clinical features.^35^ Our observation of higher baseline NfL levels predicting more rapid motor and functional progression as well as the development of postural instability mirrors several other studies.^14–17 36^ Despite only noting a trend towards baseline NfL levels being associated with cognitive progression as determined by changes in the MOCA, we noted a significant predictive capacity for earlier development of dementia. This is consistent with a previous study which suggested that NfL appears to be better at predicting the development of dementia than mild cognitive impairment.^36^ When taken together our findings of NFLs ability to predict motor, cognitive and functional progression as well as death could potentially be explained by it predicting a more malignant progression reflecting the magnitude of alpha synuclein deposition and anatomical dysfunction present.^37 38^

PD progression and prognosis can be highly variable. Several phenotypes have previously been explored with the goal of predicting future outcomes.^39^ To date, studies focusing on the potential role of NfL in predicting more severe progression phenotypes have suggested that patients with a more prominent postural instability phenotype have more substantial increases in NfL levels over time.^16 17^Our goal was to explore if NfL levels and /or genetic variables could play a role in a model which predicts PD progression in a more encompassing and practical manner that could potentially be utilised in disease modifying clinical trials. We found that baseline NfL levels could replace or complement a number of simple clinical markers previously identified to predict PD progression in a well validated model,^21^ and while we did not find that NfL alone provided significant additional value to the clinical variables previously identified, the predictive model was strongest when NfL was combined with clinical variables and patient’s genetic status. This finding highlights the potential use of combining biomarkers with clinical scales and could support its future use in randomising patients between active treatment and placebo arms in clinical trials.

The strengths of our study are its large sample size and prolonged follow-up of up to 72 months although this was only available in 23.2% of cases. We were limited by a lack of assessment in the ‘OFF’ medication state which restricts our ability to interpret NfL associations with motor progression of the dopa responsive elements of the disease and therefore limits our ability to estimate its value in clinical trial modelling where MDS-UPDRS OFF state changes may be the primary outcome. While we found no significant differences between this smaller sample of the Tracking-PD study and the broader study population, it is possible that our results might be confounded by unrecognised selection biases. We also lack neuropathological diagnostic confirmation in our cohort although our exclusion of patients with a diagnostic probability of <90% at the last available visit aimed to mitigate the potential inclusion of misdiagnosed patients.

We were able to demonstrate that the combination of serum NfL with baseline clinical outcomes and patients’ genetic status can be useful for prediction of PD progression. In the appropriate setting, this combination could potentially be used to enrich a clinical trial cohort for individuals likely to have more rapid disease progression, which might then shorten the follow-up time required to detect a disease modifying signal, or alternatively to help ensure that randomised groups are more likely to be balanced in terms of progression rates, thus facilitating detection of agents with true disease modifying properties.

## Data Availability

Data are available on reasonable request.
