# Supplementary material for: Combining biomarkers for prognostic modelling of Parkinson’s disease
Source: J Neurol Neurosurg Psychiatry. 2022 May 16;93(7):707–15. doi: 10.1136/jnnp-2021-328365 (PMC9279845; doi:10.1136/jnnp-2021-328365)
Supplement: Supplementary data [file jnnp-2021-328365supp001.pdf]

Supplementary Tables

Supplementary Table 1 Baseline characteristics of patients selected for NfL analysis compared to remaining cohort

**Mean (SD)**	**Cohort without NfL assay**	**Cohort with NfL assay**	**p-value**
**n**	1709	291	
**Age**	67.4 (9.4)	68.6 (8.8)	0.0714
**Gender, male (%)**	1110 (65.0)	185 (63.6)	0.6500
**Disease duration from diagnosis**	1.3 (0.9)	1.3 (0.9)	0.7871
**MOCA**	25.2 (3.5)	24.8 (3.8)	0.0893
**MDS-UPDRS 3**	22.8 (12.4)	23.4 (11.8)	0.2200
**H&Y**	1.7 (0.6)	1.8 (0.6)	0.1718

Abbreviations: H&Y stage Hoehn and Yahr stage; MoCA Montreal Cognitive Assessment;

MDS-UPDRS Movement Disorders Society Unified Parkinson’s disease rating scale.

Supplementary table 2 Comparison of baseline characteristics of patients with and without genetic abnormalities

Mean (SD)	GBA negative PD (n=213)	Non-GD variant PD (n=17)	GD variant PD (n=10)	Non ε4 allele PD (n=165)	Heterozygous ε4 PD (n=63)	Homozygous ε4 PD (n=8)
Age	68.6 (8.8)	67.6 (8.3)	62.0 (11.2)	69.7 (8.7)	65.4 (8.2) **	67.5 (4.9)
Gender, male (%)	132 (62.0)	15 (88.2)	6 (60.0)	112 (67.9)	36 (57.1)	5 (62.5)
Disease duration	1.3 (0.9)	1.1 (0.7)	1.1 (1.1)	1.3 (0.9)	1.3 (0.9)	1.0 (0.7)
H&Y	1.8 (0.6)	1.8 (0.6)	1.7 (0.7)	1.8 (0.6)	1.7 (0.6)	1.4 (0.6)
MDS-UPDRS 3	23.2 (11.5)	20.1 (12.6)	19.7 (16.8)	23.0 (11.8)	23.1 (10.5)	15.9 (10.4)
MOCA	25.1 (3.6)	25.7 (2.3)	24.9 (2.8)	25.2 (3.3)	24.9 (3.4)	24.0 (2.9)
SF	20.9 (6.3)	22.8 (6.6)	26.2* (4.2)	21.4 (6.3)	21.2 (6.4)	18.5 (10.4)
NfL	30.5 (17.3)	32.2 (17.4)	25.8 (17.0)	30.6 (17.2)	27.4 (13.2)	36.7 (22.9)

* GD variant PD vs GBA negative PD p<0.05, ** Heterozygous ε4 PD vs Non ε4 allele PD p<0.01

Abbreviations: GD Gaucher disease; H&Y stage Hoehn and Yahr stage; MoCA Montreal Cognitive Assessment; NfL Neurofilament light protein; PD Parkinson's Disease; MDS-UPDRS Movement Disorders Society Unified Parkinson's disease rating scale; SF Semantic fluency.

Supplementary table 3 Regression coefficients of the final combination models explored.

Model 1	Coefficient	Standard error	P value
Intercept	-5.40	2.03	
Age	0.06	0.03	0.054
Gender	-0.90	0.45	0.045
NfL	0.81	0.23	0.001
Model 2			
Intercept	-6.65	2.12	
Patient age	0.09	0.03	0.001
Gender	-0.63	0.45	0.164
UPDRS axial	0.33	0.09	<0.001
Semantic fluency	-0.10	-0.04	-0.007
Model 3			
Intercept	-3.95	2.27	
Age	0.04	0.03	0.194
Gender	-0.89	0.49	0.066
UPDRS axial	0.37	0.10	<0.001
Semantic fluency	-0.08	0.04	0.046
NfL concentration	0.82	0.26	0.001
Model 4			
Intercept	-6.20	2.71	
Age	0.07	0.04	0.044
Gender	-0.85	0.54	0.114
UPDRS Axial	0.34	0.11	0.001
Sematic Fluency	-0.09	0.04	0.041
NfL concentration	0.64	0.27	0.020

ApoE status	0.68	0.38	0.071
GBA status	0.45	0.53	0.403

Supplementary table 4 Summary of ROC analysis for models using different baseline predictive variables and comparison of models

	AUC	CI
Age+gender	0.74	0.67-0.82
1. NfL	0.79	0.72-0.85
2. UPDRS Axial	0.79	0.71-0.86
3. SF	0.78	0.71-0.85
4. GBA status	0.75	0.68-0.83
5. APOE status	0.75	0.67-0.83
6. UPDRS Axial/NfL	0.82	0.76-0.89
7. SF/NFL	0.81	0.75-0.88
8.GBA/NFL	0.79	0.72-0.86
9.ApoE/NfL	0.80	0.73-0.86
10. APOE/GBA	0.76	0.68-0.84
11. SF/UPDRS Axial	0.82	0.74-0.88
12. SF/UPDRS Axial/NfL	0.85	0.79-0.91
13. SF/UPDRS Axial/GBA	0.83	0.76-0.89
14. SF/UPDRS Axial/apoE	0.82	0.75-0.89
15. SF/UPDRS Axial/NfL/GBA	0.85	0.78-0.91
16. SF/UPDRS Axial/NfL/ApoE	0.85	0.78-0.91
17. SF/UPDRS Axial/NfL/ApoE/GBA	0.84	0.78-0.91
AUC comparison	Chi	p-value

1 vs 11	1.04	0.3073
1 vs 12	5.77	0.0163*
1 vs 15	4.68	0.0305*
1 vs 16	5.49	0.0192*
1 vs 17	3.98	0.0461*
10 vs 11	2.56	0.1098
11 vs 15	1.83	0.1761
11 vs 16	2.53	0.1118
11 vs 17	2.07	0.1505

All models incorporate age and gender as covariates

Abbreviations AUC area under the curve; NfL Neurofilament light protein; SF sematic fluency; UPDRS Unified Parkinson’s Disease Rating Scale total axial score; SF semantic fluency
